# Disparities in Infant Nutrition: WIC Participation and Rates of Breastfeeding in Florida

**DOI:** 10.3390/ijerph20115988

**Published:** 2023-05-29

**Authors:** Sarah G. Buxbaum, Olumide Arigbede, Arlesia Mathis, Fran Close, Sandra G. Suther, Elizabeth Mazzio, Remelda Saunders-Jones, Karam F. A. Soliman, Selina F. Darling-Reed

**Affiliations:** College of Pharmacy and Pharmaceutical Sciences, Institute of Public Health, Florida A & M University, Tallahassee, FL 32307, USA

**Keywords:** breastfeeding, health disparities, WIC, maternal and child health

## Abstract

Being cognizant of the pronounced health advantages of breastfeeding for both the nursing mother and her infant, the breastfeeding dyad, we examined breastfeeding rates among Floridian women who gave birth from 2012 to 2014 (*N* = 639,052). We investigated the associations between breastfeeding initiation and WIC-based breastfeeding support (the Special Supplemental Nutrition Program for Women, Infants, and Children), education level, and race and ethnicity. We compared the percentage of breastfeeding mothers between those in the WIC program and those who were not, and we compared breastfeeding rates across racial and ethnic groups. Consistent with previous reports, black newborns in this study were breastfed at lower rates than other racial groups, and WIC program participants were less likely to breastfeed than non-WIC program participants. However, by breaking down the data by education level and race, and ethnicity, we see a significantly increased rate of breastfeeding due to WIC participation for both Hispanic and black women with less than a high school education. Further, we assessed differences by insurance type, race, and WIC participation. In multivariable logistic regression, we showed that the WIC program has a significant positive impact on breastfeeding rates for all but white non-Hispanic mothers, independent of sociodemographic and geographic variables. We also note a trend of increasing breastfeeding rates over the study period (*p*-value < 0.0001), which has positive public health implications.

## 1. Introduction

In the United States (US), lower socioeconomic status is associated with lower rates of breastfeeding initiation and duration [1,2]. Along with other developed nations, such as Australia and Canada, the gap between the affluent and poor is expanding, thereby contributing to both health and racial disparities [2]. In the US, significant disparities exist in breastfeeding indices; e.g., rates of breastfeeding initiation and duration among non-Hispanic black infants are lower than those of non-Hispanic white infants, with large-scale consequences [2,3]. 

This disparity is problematic as it not only deprives the nursing mother of benefits afforded to her, such as birth spacing and the lower risk of developing breast and ovarian cancer or type 2 diabetes [4], but also creates vulnerabilities in the infant in terms of long-term emotional intelligence, orofacial structure, physiological and psychological development [5,6], cognition [7], resilience, mother-child attachment [8,9], and immune system health [2]. Worldwide, the impact of not breastfeeding results in greater morbidity and mortality associated with infectious disorders (diarrhea and respiratory), responsible for an estimated 820,000 deaths per year in children under the age of 5 [2,6]. In developed countries, the attributable childhood mortality is lower; however, a history of breastfeeding is associated with a reduction in the risk of otitis media, gastroenteritis, severe lower respiratory tract infections, atopic dermatitis, childhood asthma, childhood obesity, type I, and II diabetes, childhood leukemia, sudden infant death syndrome (SIDS), and necrotizing enterocolitis [10,11].

Many government-sponsored programs have aimed at establishing population-wide adherence to breastfeeding practices, such as Healthy People 2020 (HP2020). The HP2020 initiative sets goals to increase breastfeeding rates to a minimum of 82% of infants ever breastfed, with 61% and 34% being breastfed by 6 months and 12 months of age, respectively, and 46.2% and 25.5% of infants being exclusively breastfed to 3 and 6 months, respectively. However, the previous HP2010 agenda failed to reach its proposed quota, with the exception of 75% of new mothers who started nursing at birth [12]. Although breastfeeding rates for both black and white newborns increased over the previous decade, racial inequities still exist [13]. Asian women are the only racial/ethnic group currently reaching the Healthy People 2020 target of breastfeeding initiation, while African American women had the lowest rates of breastfeeding initiation as well as a continuation to 6 and 12 months [1].

The low rates of breastfeeding indices for African American mothers and children substantiate a long-term and complicated problem [14], subject to influence by a variety of socioecological variables such as education level, regulations (local, state, and federal), politics, and management/employer support [5,15]. In the US, mothers who breastfeed at a lower rate are more likely to be young, low-income, African American, with “divorced, single or widowed” marital status, participants in the Supplemental Nutrition Program for Women, Infants, and Children (WIC), overweight or obese before pregnancy, and more likely to report their pregnancy was unintended [16,17]. Delivery by C-section is another risk factor [18], as is preterm birth, which disproportionately affects African Americans [19]. Known hurdles to breastfeeding may be divided into two categories: microenvironmental variables, such as community environment, family environment, birthing environment, and job/work environment, and macroenvironmental ones, such as political environments at the county, state, and federal levels. Many women continue to face significant obstacles in their job and family environments [20]. Due to the confluence of these factors, even after deciding to breastfeed, mothers frequently fall short of their nursing goal [21]. Even with WIC support [22], at-risk mothers who intend to breastfeed may fail to follow up due to a lack of financial and emotional support in the community and family structure [21]. Further, multifaceted factors facing black women include a greater rate of chronic disease, stress, depression, systematic discrimination, posttraumatic stress disorder, and low-income status, all of which are associated with low breastfeeding rates [23]. Moreover, in the US, black mothers, often heading single-family households that are low-income, are disproportionately subjected to workplaces that are hostile to breastfeeding [24]. Black mothers return to work two months on average after giving birth, which is sooner than women of other racial and ethnic groupings [17,24]. These are just two of the critical obstacles encountered when returning to work, particularly for black women [5], and, more generally, breastfeeding assistance at work remains uneven for all women. As of 2011, only 23 states had established legislation addressing breastfeeding in the workplace [25], while only 37% of states required employers to provide break time or pumping sites to breastfeeding employees. As a result, many breastfeeding mothers are not protected by legislation that encourages workplace breastfeeding [26].

According to the National Conference of State Legislatures, the Fairness for Breastfeeding Mothers Act of 2019 was passed by the US Congress, creating a federal law requiring certain public buildings to provide a shielded, hygienic space other than a bathroom that contains a chair, a working surface, and an electrical outlet for use by members of the public to express milk. Currently, all fifty states, the District of Columbia, Puerto Rico, and the Virgin Islands have laws that specifically allow women to breastfeed in any public or private location. However, only 31 states, along with the District of Columbia (DC) and Puerto Rico, exempt breastfeeding from public indecency laws. A total of 30 states, including DC, and Puerto Rico have specific laws related to breastfeeding in the workplace. Four states and Puerto Rico have implemented or encouraged the development of breastfeeding awareness education campaigns [27]. 

WIC was established in 1972 [28] for the purpose of providing supplemental food, nutrition education, and healthcare referrals to pregnant, breastfeeding, and postpartum women, as well as infants and children up to the age of five [16,22]. WIC covers about half of all births in the US [29], serving to encourage mothers to exclusively breastfeed their babies during their first 6 months and to ensure breastfeeding with infant formulas and/or foods until at least 24 months [30]. Although studies have been conducted on the influence of mothers’ WIC participation on newborn health outcomes, smoking cessation, timely commencement of prenatal care, healthy weight gain [31,32], the wellbeing of mothers and children [33], and mothers’ perception and behavior towards breastfeeding [34], reports on the impact of WIC on breastfeeding practices have been inconsistent. For example, Bullinger and Gurley-Calvez (2016) found the WIC program had a statistically negligible effect on the initiation and duration of breastfeeding, with a reduction of exclusive breastfeeding by nearly half [35], stating that previous studies that described a negative association between breastfeeding and WIC participation (i.e., showing that WIC participants were less likely to breastfeed) did not control for WIC selection factors. Sonchak et al. controlled for WIC selection and found no negative effect of WIC on breastfeeding initiation [31], and Jiang et al. found that after taking sociodemographic covariates into account, there was no evidence to support the hypothesis that the WIC program itself was depressing breastfeeding initiation or duration, and to the contrary, their sibling study suggested a positive impact of the WIC program on breastfeeding [36]. Gleason et al. studied site-level characteristics of WIC programs and showed that in a study of breastfeeding mothers, breastfeeding duration was positively associated with the number of programmatic supports [37].

Despite US federal programs created to improve work and community environments to support breastfeeding, these disparities continue to exist. Supportive US federal programs include the Family Medical Leave Act (1993), the Personal Responsibility Welfare and Work Opportunity Act (1996), the Affordable Care Act (2010) [38], Healthy People (2010), and the Surgeon General’s Call to Action to Support Breastfeeding [28]. 

Poor breastfeeding outcomes are more prevalent among rural women than urban women. Several barriers have been cited for the lack of breastfeeding initiation among rural women. Kozhimannil et al. found that women giving birth tend to be younger in rural areas compared to urban areas [39]. The propensity of women to breastfeed tends to increase with maternal age. Maternal education is another barrier to poor breastfeeding outcomes in rural areas, where women are less likely to attain a college or advanced degree [40]. Access, due to greater distances to health care services, is also cited as a barrier to breastfeeding among rural women due to a lack of reliable sources to inform, educate, and support breastfeeding mothers [41,42]. The Big Bend area of Florida is a region that is generally rural, although it includes the state capital, and has a high level of infant mortality compared to the rest of the state [43]. 

For our study, trends in breastfeeding inequalities between 2012 and 2014 were examined using data from the State of Florida’s Bureau of Vital Statistics Birth Records. Specifically, the outcome studied was breastfeeding initiation, i.e., being breastfed. Given the increased breastfeeding support provided by WIC to low-income women [14,29,31], this study looked at breastfeeding inequalities, such as differences in breastfeeding initiation outcomes between WIC participants and non-WIC participants, and also assessed the change in breastfeeding initiation rates over a 3-year timeframe. This study offers a unique viewpoint on evaluating WIC program nutrition education and interventions on breastfeeding practice by comparing breastfeeding outcomes among WIC participants to nonparticipants in the entire state of Florida, taking a variety of sociodemographic factors into account. In addition, the study assesses factors that impact breastfeeding initiation in a multivariable logistic regression analysis.

## 2. Materials and Methods

This cross-sectional study used deidentified data obtained from the Florida Department of Health, Bureau of Vital Statistics, which included 639,052 women from 67 counties in the state of Florida. The data included information on women who gave birth between 2012 and 2014, both during their pregnancy and postpartum. In this data, ethnicity is defined as Hispanic. The racial groups are limited to black, white, and others. No other exclusion criteria were applied. This study examined how education and race/ethnicity affect breastfeeding health inequalities between WIC mothers and those who did not participate in WIC. We incorporated mothers’ educational levels from three race/ethnic groupings, considering Florida as a whole and then focusing more on the Big Bend counties, which are mostly rural with high poverty rates. In our analysis, the Big Bend area comprises Gadsden, Franklin, Jefferson, Leon, Liberty, Madison, Wakulla, Taylor, Dixie, Suwannee, Gilchrist, Columbia, LaFayette, Levy, Clay Hamilton, Pasco, Hernando, Citris, Baker, Union, Bradford, and Putnam counties. We removed from analysis any records with missing data in the variables we analyzed, resulting in a total of 600,026 women. SAS Studio, accessed online via SAS OnDemand for Academics, was used to conduct all analyses, testing for significant (using an alpha threshold of 0.05) relationships between WIC participation and breastfeeding initiation rates [44].

In addition, we assessed prevalence ratios for breastfeeding, comparing WIC participants to non-WIC participants to determine the impact of education, insurance, and race/ethnicity on breastfeeding initiation. Higher prevalence ratios are indicative of greater participation in breastfeeding among WIC participants. The prevalence ratio is identical to that of relative risk but does not imply a temporal aspect in cross-sectional studies such as this one [45]. Further, we conducted a multivariable logistic regression with breastfeeding as the outcome variable, stratifying the data by performing the analysis separately for each race/ethnic group. The multivariable logistic regression included the following covariates: WIC participation, education level, marital status, insurance type, location (Big Bend area or not), and also whether the mother lived in a city or not, and the birth year.

Institutional Review Board (IRB) approval was given by both the Florida Department of Health IRB (2021-473) and the Florida A&M University IRB (028-21). 

## 3. Results

### 3.1. Overview of the Data

**Figure 1:**
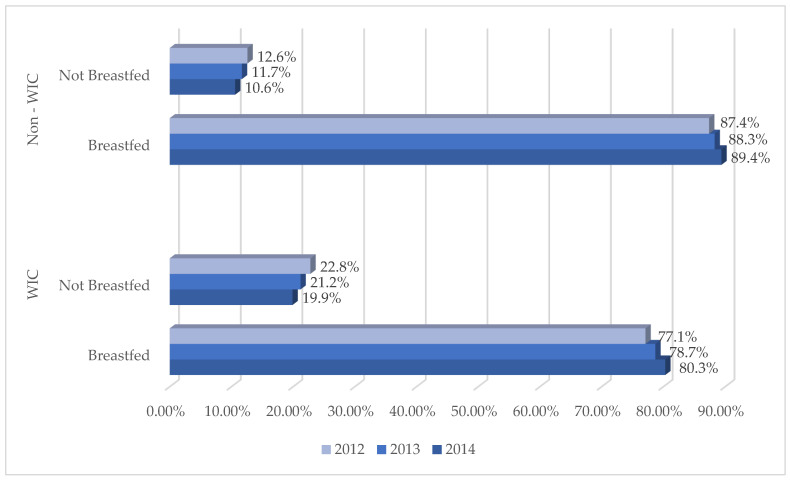
Mothers Who Breastfed by WIC Participation Status and Year.

The data comprise all women who gave birth in Florida between 2012 and 2014, the data from both during their pregnancy and after delivery (*N* = 639,052) (Table 1). In 2012, most participants (65.1%) were white (non-Hispanic or Hispanic). Black (non-Hispanic and Hispanic) participants comprised 20.9%, and other races comprised 14.7% of the population. Comparable results by race were reported in 2013 and 2014. Similarly, 54.3% of the WIC program participants were white women (Hispanic and non-Hispanic). Concerning education levels, 12.1% of the WIC participants in the Big Bend area had at least an Associate’s degree, compared to 15.5% of WIC participants outside the Big Bend area. 600,026 of the mothers had non-missing data on WIC participation. A total of 83% of the mothers breastfed (Table 2), and those in the WIC Program breastfed less frequently overall (Figure 1). Breastfeeding rates differed significantly by racial/ethnic group at every education level, with Hispanic mothers most likely to breastfeed.

### 3.2. Relationship between Race, Education, and Breastfeeding Practices among Florida Mothers

Breastfeeding behavior varies by educational level. We initially dichotomized the education level into high and low, where the high level of education category included associate, bachelor’s, master’s, and doctoral degrees and postdoctoral studies (Supplement Table S1). We then assessed the percentage of breastfeeding at each education level (Table 3) and also specifically in the Big Bend area of Florida (Table 4).

Additionally, we assessed the prevalence ratios for breastfeeding among WIC participants. Higher prevalence ratios are indicative of greater participation in breastfeeding. In Table 3, we break down the data by race and education level, showing that black Hispanic mothers with low education levels who were participating in WIC were slightly more likely to breastfeed than those of the same group who were not participating in WIC (PR 1.019; 95% CI: 1.004, 1.034). Nearly every other group was significantly less likely to breastfeed when in WIC, particularly white non-Hispanics with low education levels (PR = 0.890; 95% CI: 0.885, 0.894). For the group with low education and the “other” race, the breastfeeding rate was the same whether there was WIC participation or not. There was an increase in the breastfeeding rate over the course of the study, and in 2014, the raw number of mothers who were not in WIC and who breastfed overtook the number of mothers who were in WIC and breastfed, indicating a general cohort effect of increasing breastfeeding. 

Table 3 reports the ratios of breastfeeding rates of WIC participants relative to breastfeeding rates of non-WIC participants by race/ethnicity, and education level. Here we show that black Hispanic, black non-Hispanic, and white non-Hispanic mothers with less than a high school education are more likely to breastfeed when they are WIC participants. There was no effect of WIC on breastfeeding rates among white, non-Hispanic mothers with less than a high school education. The most dramatic difference is among black non-Hispanic mothers with less than an 8th-grade education, who were 39% more likely to breastfeed if they were in the WIC program. In the 9–12 grade education category, black Hispanic mothers and black non-Hispanic mothers were 10% and 13%, respectively, more likely to breastfeed if they were in the WIC program than those who were not. White Hispanic mothers with less than an 8th-grade education were 13% more likely to breastfeed if on WIC, but the breastfeeding rates were not significantly different in the 9–12th grade group. Table 4 also shows differences in rates of breastfeeding depending on race and ethnicity and the type of insurance used, which can be considered a proxy for socioeconomic status. 

### 3.3. Relationship between Insurance Type, WIC Participation, Race/Ethnicity, and Breastfeeding Practices among Florida Mothers

Among the mothers that self-paid for medical care, all four race/ethnic groups considered tended to have higher rates of breastfeeding among WIC participants than among non-WIC participants; however, the only statistically significant group was among white Hispanics, the largest group in this category, who were 2.26-fold more likely to breastfeed if they were WIC participants (Table 3). Among those who had private insurance, black non-Hispanic and white Hispanic mothers had statistically significant results and were 2.6-fold and 3.5-fold more likely to breastfeed if they were WIC participants, respectively. Among Medicaid patients, three groups had statistically significant results: black non-Hispanics, white Hispanics, and white non-Hispanics, with prevalence ratios of 1.43, 1.38, and 1.08, respectively. Thus, for this group, black non-Hispanics and white Hispanics had very similar increases in breastfeeding rates for WIC participants. 

### 3.4. Multivariable Logistic Regression Analysis

Adjusting for all other variables, each racial/ethnic group showed an increase in breastfeeding rates among WIC participants except for the white non-Hispanic group, who were 8% less likely to breastfeed if in the WIC program (Table 5). Single women in each group were less likely to breastfeed than married women, most dramatically for black non-Hispanic women, among whom single women were 41% less likely to breastfeed than married women. For each group, those on Medicaid were less likely to breastfeed than those with private insurance. Of the mothers who self-pay for medical care, black non-Hispanics and white mothers (both Hispanic and non-Hispanic) were significantly more likely to breastfeed. Location mattered: each group was less likely to breastfeed if in the Big Bend area. Black Hispanics, white non-Hispanics, and those in the other group were less likely to breastfeed if they lived outside a city (i.e., rural areas); in contrast, white Hispanics were more likely to breastfeed if they lived outside a city than if they lived in an urban setting. Black non-Hispanics had no difference in breastfeeding rates between urban and rural areas. 

There was a direct relationship between the amount of education and the breastfeeding rate up to at least the completion of undergraduate studies. There were enough black non-Hispanics and white non-Hispanics with doctoral/professional degrees to show a statistically significant 45% and 20% increase in breastfeeding, respectively, at the highest level of education versus a bachelor’s degree. On the other end of the spectrum, among those with less than an eighth-grade education, black non-Hispanics, and white non-Hispanics were respectively 81% and 83% less likely to breastfeed than those with a bachelor’s degree. Each group had a substantial increase in odds of breastfeeding between a high school level of education and some college, especially white Hispanics (25%). Lastly, we note that, for each group, there was an increase in the odds of breastfeeding initiation over the three-year period, ranging from 3% to 40%, depending on all other variables considered in our logistic regression model. These increases were highly statistically significant (*p*-value < 0.0001), with the exception of the smaller group of black Hispanics, for whom the increase was positive but not statistically significant. 

## 4. Discussion

We investigated the level of breastfeeding engagement in a heterogeneous population stratified by education level, insurance status, race/ethnicity, and WIC program participation. Our results demonstrate that there was a significant increase in breastfeeding by about 3% between 2012 and 2014 for all race/ethnic groups studied. We expect this cohort effect to be enhanced with the 2018 change in policy to allow doulas to be compensated by Medicaid. The WIC program appears to have successfully made a positive change for young women with low education concerning encouraging breastfeeding, with the exception of non-Hispanic white young women. The latter group could be a good target group for public health marketing to effect positive change in the state of Florida, similar to what was done in the Truth campaign against adolescent smoking [46].

Consistent with previous reports, WIC program participants were less likely to breastfeed than non-WIC program participants. 21.4% of mothers who participated in the WIC program (*n* = 319,055) between 2012 and 2014 did not breastfeed their infants, while 16.8% of all mothers in this time frame did not breastfeed. We found that among this population, and after stratifying by education, race, and insurance status, participants with “8th grade through some college credit,” black non-Hispanic race/ethnicity, and Medicaid insurance had the highest percentage (45.6%) of not breastfeeding their infants, despite being WIC participants. This finding is consistent with that of Simpson et al., who suggest that mothers who are older (over 30) and more educated are more likely to initiate breastfeeding, possibly because they have more theoretical knowledge about breastfeeding, including its health advantages for infants [47]. An alternative conclusion might be that mothers are less likely to breastfeed if they lack familial/spousal support as well as time to breastfeed due to work requirements, and/or that there is a lack of knowledge of supportive policies and legislation [19,48]. 

Despite numerous breastfeeding programs, supportive legislation, and general success in increasing US breastfeeding rates over the past couple of years, black mothers have the lowest breastfeeding rates [5,49]. To address this health disparity, interventions are required to remove obstacles to black women breastfeeding. These include but are not limited to the following: encouraging breastfeeding time-off at the workplace, some 30 min, for breastfeeding employees; and providing consistent breastfeeding education to minority communities, clearly stating the reasons for its importance [50]. 

Geographic location is an important factor influencing health outcomes, and we found this to be true for breastfeeding rates in Florida. We examined the breastfeeding rates of mothers in the Big Bend counties and saw the same trends as in the state-wide data; however, with the lower counts, the prevalence ratios for mothers were not significantly different for WIC participants than for non-WIC participants, with the only exception being white non-Hispanic mothers with a doctorate or professional degree. In that group, WIC participants were 8% more likely to breastfeed than non-WIC participants. In the multivariable logistic regression, we found that mothers in the Big Bend area were significantly less likely to breastfeed compared to those not in this area and that mothers were 13 to 30% more likely to breastfeed if they were WIC participants, independent of all socioeconomic factors studied—with the exception of the white non-Hispanic women, who had 8% lower odds of breastfeeding if they were WIC participants compared to those not in the WIC program. 

We hypothesized that women in the city would have more access to resources and thus be more encouraged to breastfeed in urban areas; however, in the multivariable logistic regression model results, we saw that while this was true for mothers who identified as black Hispanic, white non-Hispanic, and mothers with “other” races, in fact, white Hispanic women had greater odds of breastfeeding if they did not live in the city, and there was no significant difference for black non-Hispanic women. Thus, it appears that there are influences in the cities that dissuade or prevent white Hispanic women from breastfeeding that need to be addressed. Hispanics in Florida are a heterogeneous group, and there may be additional cultural influences within this group in rural vs. urban areas that could be investigated. The fact that there is no significant difference for black non-Hispanic women, whether urban or rural, despite the greater availability of resources in cities, suggests that outreach to this group needs to be improved.

In order to reach the sustainable development goals (SDGs) and the US Healthy People 2030 goals, infant mortality rates must be reduced. A plethora of benefits exist for mothers and children. One important approach to improving infant health is through programs that support breastfeeding, such as the WIC program. Although most Florida mothers breastfeed their infants, there are several variables that influence this practice in addition to WIC participation, including income, race, ethnicity, geography, and degree of education. A multilevel set of interventions is required to successfully effect change in breastfeeding rates: family support, especially from immediate relatives, including grandmothers; other in-person support such as peer counseling; advice to breastfeed from their health provider; employer support; support for breastfeeding at the hospital with the encouragement of breastfeeding with skin-to-skin contact; and lactation consultants [5]. Further, it is important in public health media campaigns that women see other women who look similar to themselves [48].

By using birth data from the Florida Department of Health’s Bureau of Vital Statistics, we were able to obtain a high level of granularity in this dataset. A limitation was that we could not assess in this dataset the influence of community-level efforts that impact the efficacy of breastfeeding education. In future work, we will be adding data from more recent years to our study.

## 5. Conclusions

The health disparities that are seen in breastfeeding reflect wider health disparities. Maternal and child health is significantly influenced by factors such as race and ethnicity, education, insurance, and many more. We must learn more about how these factors interact and their potential impact on public health. Furthermore, our analysis shows that there are complex patterns associated with breastfeeding status, WIC participation, and risk factors for mothers. To reduce disparities associated with breastfeeding, major efforts are still needed to promote breastfeeding initiation and duration rates in the US. Our research indicates that some racial/ethnic minority women may have experienced more trouble initiating and maintaining breastfeeding because of factors that contribute to disparities. In this granular study of breastfeeding in Florida, we found significant evidence of a positive impact of WIC participation, particularly for minority women with low education levels.

## Figures and Tables

**Table 1:** Characteristics of the Study Population.

Characteristics	Black Hispanic	Black Non-Hispanic	White Hispanic	White Non-Hispanic	Other	Total
	*n* (%)	*n* (%)	*n* (%)	*n* (%)	*n* (%)	*N* (%)
Breastfeeding						
Yes	23,506 (87.1)	81,364 (70.1)	98,661 (89.3)	227,754 (84.3)	67,862 (88.8)	449,147 (83.2)
No	3473 (12.9)	34,729 (29.9)	11,798 (10.7)	42,301 (15.7)	8578 (11.2)	100,879 (16.8)
WIC Participation						
Yes	20,877 (77.4)	82,891 (71.4)	64,534 (58.4)	106,784 (39.5)	43,969 (57.5)	319,055 (53.2)
No	6102 (22.6)	33,202 (28.6)	45,925 (41.6)	163,271 (60.5)	32,471 (42.5)	280,971 (46.8)
Education						
Less than 8th grade	1069 (4.0)	1184 (1.0)	6986 (6.3)	2453 (0.9)	7633 (10.0)	19,325 (3.2)
9th–12th grade; no diploma	4050 (15.0)	18,704 (16.1)	12,732 (11.5)	21,284 (7.9)	9057 (11.8)	65,827 (11.0)
High school graduate or GED	10,196 (37.8)	46,889 (40.4)	36,158 (32.7)	71,820 (26.6)	21,852 (28.6)	186,915 (31.2)
Some college credit, no degree	5331 (19.8)	25,884 (22. 3)	18,377 (16.6)	55,395 (20.5)	12,017 (15. 7)	117,004 (19.5)
Associate’s degree	2936 (10.9)	8906 (7.7)	11,643 (10.5)	28,957 (10.7)	6326 (8.3)	58,768 (9.8)
Bachelor’s degree	2387 (8.8)	9075 (7.8)	16,284 (14.7)	59,814 (22.2)	12,233 (16.0)	99,793 (16.6)
Master’s degree	717 (2.7)	3935 (3.4)	6029 (5.5)	22,882 (8.5)	4959 (6.5)	38,522 (6.4)
Doctorate or professional degree	158 (0.6)	820 (0.7)	1656 (1.5)	6331 (2.3)	1846 (2.4)	10,811 (1.8)
Others	135 (0.5)	696 (0.6)	594 (0.5)	1119 (0.4)	517 (0.7)	3061 (0.5)
Marital Status						
Single	12,953 (48.0)	87,728 (75.6)	53,889 (48.8)	101,740 (37.7)	33,404 (43.7)	289,714 (48.3)
Married	14,026 (52.0)	28,365 (24.4)	56,570 (51.2)	168,315 (62.3)	43,036 (56.3)	310,312 (51.7)
Insurance						
Medicaid	16,858 (62.5)	86,243 (74.3)	58,513 (53.0)	116,620 (43.2)	37,155 (48.6)	315,389 (52.6)
Private Insurance	5739 (21.3)	62,267 (22.6)	38,403 (34.8)	143,478 (53.1)	26,775 (35. 0)	240,662 (40.1)
Self-Pay	4382 (16.2)	3583 (3.1)	13,543 (12.3)	9957 (3.7)	12,510 (16.4)	43,975 (7.3)
Geography/Location						
Big Bend Counties	26,710 (99.0)	8295 (7.2)	1604 (1.4)	22,992 (8.5)	2619 (3.4)	62,220 (10.4)
Other Counties	269 (1.0)	107,798 (92.8)	108,855 (98.6)	247,063 (91.5)	73,821 (96.6)	537,806 (89.6)
In City						
Yes	24,052 (89.2)	97,836 (84.3)	71,355 (64.6)	181,611 (67.3)	62,797 (82.2)	437,651 (72.9)
No	2927 (10.8)	18,257 (15.7)	39,104 (35.4)	88,444 (32.8)	13,643 (17.8)	162,375 (27.1)
Birth Year						
2012	9044 (33.5)	39,421 (34.0)	34,929 (31.6)	89,257 (33.0)	27,109 (35.5)	199,760 (33.3)
2013	8938 (33.1)	38,564 (33.2)	36,634 (33.2)	90,223 (33.4)	25,156 (32.9)	199,515 (33.2)
2014	8997 (33.4)	38,108 (32.8)	38,896 (35.2)	90,575 (33.5)	24,175 (31.6)	200,751 (33.5)

**Table 2:** Percentage of Mothers Who Initiated Breastfeeding, by Year.

Breastfeeding Status		Year			
		2012	2013	2014	Total
No	N	36,344	33,591	30,944	100,879
	%	18.2%	16.8%	15.4%	16.8%
Yes	N	163,416	165,924	169,807	499,147
	%	81.8%	83.2%	84.6%	83.2%
Total	N	199,760	199,515	200,751	600,026

**Table 3:** Breastfeeding Prevalence Ratios by Race/ethnicity, and Education Level and Insurance Type in Florida.

Race/Ethnicity					
Education Level/Degree		Black Hispanic PR [95% CI] * (*n*)	Black non-Hispanic PR [95% CI] (*n*)	White Hispanic PR [95% CI] (*n*)	White non-Hispanic PR [95% CI] (*n*)
	8th grade or less	1.01 [0.93–1.09] (1069)	1.39 [1.18–1.64] (1184)	1.13 [1.09–1.17] (6986)	1.00 [0.93–1.09] (2453)
	9th–12th grade, no diploma	1.10 [1.05–1.15] (4050)	1.13 [1.10–1.18] (18704)	1.02 [0.99–1.05] (12732)	0.97 [0.95–0.99] (21284)
	High school	1.01 [0.99–1.03] (10196)	1.01 [0.90–1.03] (46889)	0.98 [0.97–0.99] (36158)	0.91 [0.90–0.92] (71820)
	Some college credit, no degree	1.01 [0.99–1.04] (5331)	0.97 [0.95–0.98] (25884)	0.98 [0.97–0.99] (18377)	0.92 [0.91–0.93] (55395)
	Associate’s degree	1.0 [0.97–1.02] (2963)	0.95 [0.93–0.97] (8906)	0.98 [0.97–0.99] (11643)	0.94 [0.93–0.95] (28957)
	Bachelor’s degree	0.98 [0.96–1.00] (2387)	0.95 [0.94–0.96] (9075)	0.98 [0.97–0.99] (16284)	0.98 [0.97–0.99] (59814)
	Master’s degree	1.0 [0.96–1.05] (717)	0.95 [0.92–0.97] (3935)	0.97 [0.94–0.99] (6029)	0.99 [0.97–1.00] (22882)
	Doctorate or professional degree	0.92 [0.82–1.04] (158)	0.97 [0.90–1.04] (820)	1.01 [0.98–1.06] (656)	1.01 [1.00–1.05] (6331)
Insurance Status	Medicaid	0.92 [0.48–1.79] (592)	1.43 [1.26–1.62] (1071)	1.38 [1.15–1.66] (3232)	1.08 [0.97–1.21] (2212)
	Private	1.54 [0.50–4.76] (61)	2.63 [1.06–6.48] (66)	3.46 [1.51–7.91] (202)	0.71 [0.45–1.11] (158)
	Self-pay	1.10 [0.63–1.92] (416)	1.41 [0.44–4.56] (47)	2.26 [1.87–2.74] (3552)	1.99 [0.89–4.44] (83)

* The denominator in the prevalence ratio is the prevalence risk of the reference group, i.e., the prevalence risk for the non-WIC participant counterparts for each race/ethnicity and education or insurance level.

**Table 4:** Breastfeeding Prevalence Ratios by Race/ethnicity and Education Level in Big Bend Counties.

Race/Ethnicity					
Education Level/Degree		Black Hispanic PR [95% CI] **	Black Non-Hispanic PR [95% CI]	White Hispanic PR [95% CI]	White Non-Hispanic PR [95% CI]
	8th grade or less	NA*	1.74 [0.77–3.93]	0.92 [0.70–1.21]	1.13 [0.88–1.45]
	9th–12th grade, no diploma	2.22 [0.44–11.19]	0.94 [0.79–1.11]	1.05 [0.85–1.30]	1.05 [0.97–1.14]
	High school	0.96 [0.71–1.31]	0.96 [0.87–1.06]	0.99 [0.89–1.09]	0.90 [0.87–0.92]
	Some college credit, no degree	0.89 [0.73–1.09]	0.96 [0.90–1.03]	1.00 [0.91–1.11]	0.93 [0.90–0.95]
	Associate degree	0.73 [0.47–1.12]	0.90 [0.81–1.00]	0.95 [0.83–1.09]	0.88 [0.85–0.92]
	Bachelor’s degree	0.86 [0.65–1.14]	0.93 [0.87–0.99]	0.90 [0.76–1.05]	0.95 [0.91–0.99]
	Master’s degree	NA *	0.86 [0.76–0.97]	1.08 [0.71–1.65]	0.91 [0.82–1.01]
	Doctorate or professional degree	NA *	1.00 [0.76–1.32]	NA *	1.08 [1.05–1.12]

* The sample size is less than 20. NA—not available. ** The denominator in the prevalence ratio is the prevalence risk of the reference group, i.e., the prevalence risk for the non-WIC participant counterparts for each race/ethnicity and education level.

**Table 5:** Multivariable Logistic Regression on Breastfeeding Status by Race & Ethnicity.

Characteristics	Black Non-Hispanic	Black Hispanic	White Non-Hispanic	White Hispanic	Other
	OR [95% CI]	OR [95% CI]	OR [95% CI]	OR [95% CI]	OR [95% CI]
***N = 600,026***	*N* = 116,093	*N* = 26,979	*N* = 270,055	*N* = 110,459	*N* = 76,440
**WIC status**					
Yes	1.22 [1.18, 1.26]	1.23 [1.12, 1.36]	0.92 [0.90, 0.95]	1.13 [1.07, 1.19]	1.30 [1.22, 1.27]
No	Reference	Reference	Reference	Reference	Reference
*p*-value	<0.0001 *	<0.0001 *	<0.0001 *	<0.0001 *	<0.0001 *
**Education**					
Less than 8th grade	0.19 [0.16, 0.22]	0.39 [0.31, 0.49]	0.17 [1.09, 1.26]	0.29 [0.26, 0.32]	0.24 [0.21, 0.27]
9th–12th grade; no diploma	0.19 [0.18, 0.21]	0.42 [0.35, 0.51]	0.22 [0.21, 0.23]	0.32 [0.29, 0.35]	0.29 [0.26, 0.32]
High School Grad/GED	0.28 [0.26, 0.30]	0.49 [0.41, 0.58]	0.37 [0.35, 0.38]	0.44 [0.40, 0.48]	0.39 [0.35, 0.43]
Some college credits, no degree	0.47 [0.44, 0.51]	0.63 [0.52, 0.76]	0.53 [0.51, 0.55]	0.69 [0.63, 0.75]	0.58 [0.52, 0.65]
Associate’s degree	0.62 [0.57, 0.68]	0.65 [0.53, 0.79]	0.65 [0.62, 0.69]	0.70 [0.64, 0.78]	0.69 [0.61, 0.79]
Bachelor’s degree	Reference	Reference	Reference	Reference	Reference
Master’s degree	1.03 [0.90, 1.18]	0.84 [0.62, 1.17]	1.07 [1.00, 1.14]	0.99 [0.87, 1.14]	1.11 [0.94, 1.31]
Doctorate or professional degree	1.45 [1.08, 2.01]	0.83 [0.47, 1.61]	1.20 [1.06, 1.35]	0.88 [0.71, 1.11]	1.25 [0.97, 1.65]
Others	0.23 [0.19, 0.27]	0.26 [0.17, 0.41]	0.35 [0.30, 0.41]	0.58 [0.44, 0.78]	0.28 [0.22, 0.36]
*p*-value	<0.0001 *	<0.0001 *	<0.0001 *	<0.0001 *	<0.0001 *
**Marital Status**					
Single	0.59 [0.57, 0.61]	0.68 [0.63, 0.73]	0.76 [0.74, 0.78]	0.80 [0.76, 0.83]	0.73 [0.70, 0.77]
Married	Reference	Reference	Reference	Reference	Reference
*p*-value	<0.0001 *	<0.0001 *	<0.0001 *	<0.0001 *	<0.0001 *
**Insurance**					
Medicaid	0.58 [0.56, 0.61]	0.73 [0.65, 0.81]	0.63 [0.61, 0.65]	0.72 [0.67, 0.76]	0.61 [0.57, 0.66]
Self-Pay	1.38 [1.25, 1.52]	0.93 [0.81, 1.07]	1.17 [1.09, 1.26]	1.30 [1.20, 1.41]	1.05 [0.96, 1.15]
Private	Reference	Reference	Reference	Reference	Reference
*p*-value	<0.0001 *	<0.0001 *	<0.0001 *	<0.0001 *	<0.0001 *
**Bigbend status**					
In Big Bend	0.55 [0.52, 0.57]	0.61 [0.45, 0.84]	0.65 [0.63, 0.67]	0.56 [0.50, 0.64]	0.62 [0.56, 0.69]
Not in Big Bend	Reference	Reference	Reference	Reference	Reference
*p*-value	<0.0001 *	0.0018 *	<0.0001 *	<0.0001 *	<0.0001 *
**Geography**					
City	Reference	Reference	Reference	Reference	Reference
Not in city	0.98 [0.95, 1.02]	0.78 [0.70, 0.87]	0.95 [0.93, 0.97]	1.23 [1.18, 1.28]	0.73 [0.69, 0.77]
*p*-value	0.3626	<0.0001 *	<0.0001 *	<0.0001 *	<0.0001 *
**Birth Year**					
2012	Reference	Reference	Reference	Reference	Reference
2013	1.11 [1.08, 1.15]	1.01 [0.93, 1.10]	1.10 [1.07, 1.13]	1.11 [1.06, 1.17]	1.10 [1.04, 1.16]
2014	1.24 [1.20, 1.28]	1.03 [0.94, 1.13]	1.19 [1.16, 1.23]	1.40 [1.33, 1.46]	1.12 [1.06, 1.19]
*p*-value	<0.0001 *	0.7955	<0.0001 *	<0.0001 *	<0.0001 *

* statistically significant at an α level of 0.05.

## Data Availability

The data are not publicly available but may be requested from the Florida Department of Health.

## Supplementary Materials

The following supporting information can be downloaded at: https://www.mdpi.com/article/10.3390/ijerph20115988/s1, Table S1: Prevalence Ratios for Breastfeeding by Racial/Ethnic Groups.

## References

[1] Jones K.M., Power M.L., Queenan J.T., Schulkin J. (2015). Racial and ethnic disparities in breastfeeding. Breastfeed Med..

[2] Victora C.G., Bahl R., Barros A.J.D., França G.V.A., Horton S., Krasevec J., Murch S., Sankar M.J., Walker N., Rollins N.C. (2016). Breastfeeding in the 21st century: Epidemiology, mechanisms, and lifelong effect. Lancet.

[3] Anstey E.H., Chen J., Elam-Evans L.D., Perrine C.G. (2017). Racial and Geographic Differences in Breastfeeding—United States, 2011–2015. Morb. Mortal. Wkly. Rep..

[4] Dieterich C.M., Felice J.P., O’Sullivan E., Rasmussen K.M. (2013). Breastfeeding and health outcomes for the mother-infant dyad. Pediatr. Clin. N. Am..

[5] Johnson A., Kirk R., Rosenblum K.L., Muzik M. (2015). Enhancing breastfeeding rates among African American women: A systematic review of current psychosocial interventions. Breastfeed Med..

[6] Van Breevoort D., Tognon F., Beguin A., Ngegbai A.S., Putoto G., van den Broek A. (2021). Determinants of breastfeeding practice in Pujehun district, southern Sierra Leone: A mixed-method study. Int. Breastfeed J..

[7] Horta B.L., Loret de Mola C., Victora C.G. (2015). Breastfeeding and intelligence: A systematic review and meta-analysis. Acta Paediatr..

[8] Kramer M.S., Fombonne E., Igumnov S., Vanilovich I., Matush L., Mironova E., Bogdanovich N., Tremblay R.E., Chalmers B., Zhang X. (2008). Effects of prolonged and exclusive breastfeeding on child behavior and maternal adjustment: Evidence from a large, randomized trial. Pediatrics.

[9] Krol K.M., Grossmann T. (2018). Psychological effects of breastfeeding on children and mothers. Bundesgesundheitsblatt Gesundh. Gesundh..

[10] Bartick M., Reinhold A. (2010). The burden of suboptimal breastfeeding in the United States: A pediatric cost analysis. Pediatrics.

[11] Ip S., Chung M., Raman G., Chew P., Magula N., DeVine D., Trikalinos T., Lau J. (2007). Breastfeeding and maternal and infant health outcomes in developed countries. Evid. Rep. Technol. Assess.

[12] Scanlon K., Grummer-Strawn L., Li R., Chen J., Molinari N., Perrine C. (2010). Racial and Ethnic Differences in Breastfeeding Initiation and Duration, by State—National Immunization Survey, United States, 2004–2008.

[13] Li R., Perrine C.G., Anstey E.H., Chen J., MacGowan C.A., Elam-Evans L.D. (2019). Breastfeeding Trends by Race/Ethnicity among US Children Born from 2009 to 2015. JAMA Pediatr..

[14] Rasmussen K., Latulippe M., Yaktine A., Committee to Review WIC Food Packages, Food and Nutrition Board, Institute of Medicine, National Academies of Sciences, Engineering, and Medicine (2016). Promotion, Motivation, and Support of Breastfeeding with the WIC Food Packages. Review of WIC Food Packages: Proposed Framework for Revisions: Interim Report.

[15] Williams D.R., Mohammed S.A., Leavell J., Collins C. (2010). Race, socioeconomic status, and health: Complexities, ongoing challenges, and research opportunities. Ann. N. Y. Acad. Sci..

[16] Finer S., Henshaw S. (2006). Disparities in rates of unintended pregnancy In the United States 1994 and 2001. Perspect. Sex. Reprod. Health.

[17] Beauregard J.L., Hamner H.C., Chen J., Avila-Rodriguez W., Elam-Evans L.D., Perrine C.G. (2019). Racial Disparities in Breastfeeding Initiation and Duration Among U.S. Infants Born in 2015. Morb. Mortal. Wkly. Rep..

[18] Evans K.C., Evans R.G., Royal R., Esterman A.J., James S.L. (2003). Effect of caesarean section on breast milk transfer to the normal term newborn over the first week of life. Arch. Dis. Child. Fetal Neonatal Ed..

[19] Tran V., Reese Masterson A., Frieson T., Douglass F., Perez-Escamilla R., O’Connor Duffany K. (2023). Barriers and facilitators to exclusive breastfeeding among Black mothers: A qualitative study utilizing a modified Barrier Analysis approach. Matern. Child. Nutr..

[20] Hawkins S.S., Stern A.D., Gillman M.W. (2013). Do state breastfeeding laws in the USA promote breast feeding?. J. Epidemiol. Community Health.

[21] Lind J.N., Perrine C.G., Li R.W., Scanlon K.S., Grummer-Strawn L.M. (2014). Racial disparities in access to maternity care practices that support breastfeeding—United States, 2011. Morb. Mortal. Wkly. Rep..

[22] Florida Health Women Women, Infants, and Children (WIC). https://www.floridahealth.gov/programs-and-services/wic/index.html.

[23] Canady R.B., Bullen B.L., Holzman C., Broman C., Tian Y. (2008). Discrimination and symptoms of depression in pregnancy among African American and White women. Womens Health Issues.

[24] Ogbuanu C., Glover S., Probst J., Hussey J., Liu J. (2011). Balancing work and family: Effect of employment characteristics on breastfeeding. J. Hum. Lact..

[25] Murtagh L., Moulton A.D. (2011). Working mothers, breastfeeding, and the law. Am. J. Public Health.

[26] Nguyen T.T., Hawkins S.S. (2013). Current state of US breastfeeding laws. Matern. Child. Nutr..

[27] Breastfeeding State Laws. Ncsl.org/health/breastfeeding-state-laws.

[28] U.S. Department of Health and Human Services [DHHS] The Surgeon General’s Call to Action to Support Breastfeeding. https://www.cdc.gov/breastfeeding/resources/calltoaction.htm.

[29] USDA Food and Nutrition Service Breastfeeding is a Priority in the WIC Program. https://www.fns.usda.gov/wic/breastfeeding-priority-wic-program.

[30] WIC Breastfeeding Support (USDA) Explore the Stages of Breastfeeding [Recorded by WIC Breastfeeding Support]. https://wicbreastfeeding.fns.usda.gov/.

[31] Sonchak L. (2017). The impact of WIC on breastfeeding initiation and gestational weight gain: Case study of South Carolina Medicaid mothers. Child. Youth Serv. Rev..

[32] Testa A., Jackson D.B. (2021). Race, ethnicity, WIC participation, and infant health disparities in the United States. Ann. Epidemiol..

[33] Black M.M., Cutts D.B., Frank D.A., Geppert J., Skalicky A., Levenson S., Casey P.H., Berkowitz C., Zaldivar N., Cook J.T. (2004). Special Supplemental Nutrition Program for Women, Infants, and Children participation and infants’ growth and health: A multisite surveillance study. Pediatrics.

[34] Nouer S.S., Ware J.L., Baldwin K.M., Hare M.E. (2015). Changes in Breastfeeding Attitudes in a Metropolitan Community in Tennessee. J. Hum. Lact..

[35] Bullinger L.R., Gurley-Calvez T. (2016). WIC participation and maternal behavior: Breastfeeding and work leave. Contemp. Econ. Policy.

[36] Jiang M., Foster E.M., Gibson-Davis C.M. (2010). The effect of WIC on breastfeeding: A new look at an established relationship. Child. Youth Serv. Rev..

[37] Gleason S., Wilkin M.K., Sallack L., Whaley S.E., Martinez C., Paolicelli C. (2020). Breastfeeding Duration Is Associated With WIC Site-Level Breastfeeding Support Practices. J. Nutr. Educ. Behav..

[38] Kapinos K.A., Bullinger L., Gurley-Calvez T. (2017). Lactation Support Services and Breastfeeding Initiation: Evidence from the Affordable Care Act. Health Serv. Res..

[39] Kozhimannil K.B., Interrante J.D., Henning-Smith C., Admon L.K. (2019). Rural-Urban Differences In Severe Maternal Morbidity And Mortality in The US, 2007–2015. Health Aff..

[40] Sparks P.J. (2010). Rural-urban differences in breastfeeding initiation in the United States. J. Hum. Lact..

[41] Ertem I.O., Votto N., Leventhal J.M. (2001). The timing and predictors of the early termination of breastfeeding. Pediatrics.

[42] Gamm L., Hutchison L., Bellamy G., Dabney B.J. (2002). Rural healthy people 2010: Identifying rural health priorities and models for practice. J. Rural. Health.

[43] Wimberley D.W. (2010). Quality of life trends in the Southern Black Belt 1980-2005: A research note. J. Rural. Soc. Sci..

[44] SAS Institute (2023). SAS OnDemand for Academics: Studio.

[45] McNutt L.A., Wu C., Xue X., Hafner J.P. (2003). Estimating the relative risk in cohort studies and clinical trials of common outcomes. Am. J. Epidemiol..

[46] Zucker D., Hopkins R.S., Sly D.F., Urich J., Kershaw J.M., Solari S. (2000). Florida’s “truth” campaign: A counter-marketing, anti-tobacco media campaign. J. Public Health Manag. Pract..

[47] Simpson D.A., Quigley M.A., Kurinczuk J.J., Carson C. (2019). Twenty-five-year trends in breastfeeding initiation: The effects of sociodemographic changes in Great Britain, 1985–2010. PLoS ONE.

[48] Ware J.L., Mzayek F., Levy M. (2016). Lessons Learned in a Breastfeeding Media Campaign. Breastfeed Med..

[49] Chiang K.V., Li R., Anstey E.H., Perrine C.G. (2021). Racial and Ethnic Disparities in Breastfeeding Initiation horizontal line United States, 2019. Morb. Mortal. Wkly. Rep..

[50] Chapman D.J., Perez-Escamilla R. (2012). Breastfeeding among minority women: Moving from risk factors to interventions. Adv. Nutr..

