# Supplementary material for: Disparities in Infant Nutrition: WIC Participation and Rates of Breastfeeding in Florida
Source: Int J Environ Res Public Health. 2023 May 29;20(11):5988. doi: 10.3390/ijerph20115988 (PMC10253221; doi:10.3390/ijerph20115988)
Supplement: Supplementary file 1 [file ijerph-20-05988-s001.zip › ijerph-2224643-supplementary.pdf]

Table S1 Supplement reports the ratios of breastfeeding rates of WIC participants relative to breastfeeding rates of non-WIC participants by race/ethnicity, and education level. Here we show that black Hispanic, black non-Hispanic, and white non-Hispanic mothers with less than a high school education (low) are more likely to breastfeed when they are WIC participants. There was no effect of WIC on breastfeeding rates among white, non-Hispanic mothers with less than a high school education. The most dramatic difference is among black non-Hispanic mothers with less than an 8th-grade education, who were 39% more likely to breastfeed if they were in the WIC program. In the 9-12 grade education category, black Hispanic mothers and black non-Hispanic mothers were 10% and 13%, respectively, more likely to breastfeed if they were in the WIC program than those who were not. White Hispanic mothers with less than an 8th-grade education were 13% more likely to breastfeed if in WIC, but the breastfeeding rates were not significantly different in the 9-12th grade group. Table 3 also shows differences in rates of breastfeeding depending on race and ethnicity and the type of insurance used, which can be considered a proxy for socioeconomic status.

Table S1 Supplement. Prevalence Ratios for Breastfeeding by Racial/Ethnic Groups

**Prevalence Ratios for Breastfeeding**					
**Race/Ethnicity**	**Education Level**	**Sample Size (N)**	**Prevalence Ratio**	**95% Confidence Intervals**	
				**Lower Limit**	**Upper Limit**
**Black Hispanic**	Low	21740	1.019	1.004	1.034
	High	6572	0.979	0.965	0.994
**Black non-Hispanic**	Low	95744	0.988	0.977	0.998
	High	24743	0.922	0.913	0.931
**White Hispanic**	Low	76518	0.978	0.973	0.984
	High	36818	0.972	0.966	0.978
**White non-Hispanic**	Low	157529	0.890	0.885	0.894
	High	126148	0.944	0.939	0.950
**Other**	Low	55708	0.995	0.988	1.002
	High	28017	0.983	0.977	0.990
